# The relationships between the family impact and distress of the coronavirus disease-19 pandemic, parent insomnia, infant temperamental negative affectivity, and parent-reported infant sleep: a path analysis

**DOI:** 10.1093/sleepadvances/zpae061

**Published:** 2024-08-14

**Authors:** Nana Jiao, Keenan A Pituch, Megan E Petrov

**Affiliations:** Edson College of Nursing and Health Innovation, Arizona State University, Phoenix, AZ, USA; Edson College of Nursing and Health Innovation, Arizona State University, Phoenix, AZ, USA; Edson College of Nursing and Health Innovation, Arizona State University, Phoenix, AZ, USA

**Keywords:** COVID-19, pandemics, parental sleep, stress, temperament, infant sleep, mediation

## Abstract

**Study Objectives:**

The coronavirus disease 2019 (COVID-19) pandemic impact on infant sleep (IS) is understudied. The purpose of this study was to examine the relationships between family impact and distress from COVID-19 pandemic stressors, parental insomnia symptoms, infant temperamental negative affectivity, and parent-reported IS.

**Methods:**

Parents from the Phoenix metropolitan area with a full-term healthy infant (<1 year) were recruited from February 27, 2021, to August 7, 2021. A sample of 70 parents (baby age 5.5 ± 3.5 months; parental age: 31.7 ± 5.0 years) completed the COVID-19 Exposure and Family Impact Survey (CEFIS) Impact and Distress scales, the Insomnia Severity Index (ISI), the Infant Behavioral Questionnaire-Revised Negative Affectivity subscale (IBQ-R-NA), and the Brief Infant Sleep Questionnaire-Revised (BISQ-R). Based on the transactional model of IS, path analyses were conducted to identify the direct effect of CEFIS scores and the indirect effects of parental ISI and infant IBQ-R-NA scores on BISQ-R scores.

**Results:**

The parent sample was predominantly female (94.3%), white (72.9%), and married or in a domestic partnership (98.6%). Although COVID-19 pandemic impact and distress were not directly related to parent-reported IS, pandemic distress was negatively related to parent-reported IS indirectly through infant negative affectivity, including BISQ-R total score (β = −0.14, 95% CI [−0.32, −0.01]) and IS subscale score (β = −0.12, 95% CI [−0.27, −0.01]).

**Conclusions:**

Heightened COVID-19 pandemic family distress was related to poorer parent-reported IS through greater parent-reported infant negative affectivity, suggesting the importance of addressing family stress and emotional regulation during crises.

**Statement of Significance**

This study was among the few to investigate the associations between coronavirus disease 2019 pandemic family impact, related stress, and parent-reported infant sleep (IS). The findings underscore the importance of addressing maternal anxiety or depression and enhancing infant temperamental negative affectivity during crises to improve IS quality (e.g. sleep onset latency, number and duration of night wakings, longest stretch of sleep, and total night sleep).

The transactional model of infant sleep (IS) posits that IS is influenced by bidirectional exchanges between (1) parenting factors, including parental behaviors, cognitions, and emotions, through parental interactive behaviors (e.g. bedtime interactions) and (2) intrinsic infant characteristics, including health, maturity, and temperament [1]. Parenting and infant factors can be influenced by their culture, environmental context (e.g. coronavirus disease 2019 [COVID-19] pandemic impact), and family context (e.g. family stress) [1]. Before the COVID-19 pandemic, the greater literature found that poor parental sleep and greater parental stress were linked to parent-reported IS problems [2, 3] and also associated with greater infant temperamental negative affectivity [4, 5]. Furthermore, many longitudinal and cross-sectional studies found that negative infant temperament was associated with parent-reported IS problems, including shorter sleep duration, longer sleep latency, and more night wakings and wakefulness [6–11]. Infant temperamental negative affectivity at the age of 9 months was also positively related to more frequent nighttime awakenings at the age of 2 years [12], suggesting that negative infant temperament predicts acute and future sleep problems. However, it is less known how the impact of the COVID-19 pandemic on families with infants may have affected these relationships.

The COVID-19 pandemic altered many families’ sleep–wake patterns, which was likely in part due to major disruptions (e.g. social isolation, childcare closure, unemployment, and limited access to resources) to daily life [13–15], and heightened family chaos and parent–child conflicts [14]. A recent systematic review and meta-analysis revealed that the prevalence of sleep problems among children and adolescents during the COVID-19 pandemic was 54%, with 27% experiencing worse parent-reported sleep quality and 16% reduced parent-reported sleep duration [16]. However, research presents limited results regarding IS and associated factors during the pandemic [17–20]. Compared to prepandemic, during the pandemic, infants experienced longer auto-videosomnography assessed nighttime sleep durations, earlier bedtimes, attenuated weekday–weekend differences in sleep onset and midpoint times, yet later morning rise times, and increased sleep latency and nocturnal wakefulness [17, 18]. Factors associated with parent-reported IS examined during the pandemic included infant and parent age, household income, room sharing, race/ethnicity [19, 20], and parental distress [21, 22].

Among parents, a later bedtime and a delayed sleep midpoint were reported during the pandemic than prepandemic [23–25]. A higher clinical prevalence of parental insomnia symptoms (Insomnia Severity Index [ISI] >15) was also found: 23% during the pandemic versus 11% before the pandemic [26]. These parental sleep problems appeared to persist over an extended period despite increased vaccination uptake [27, 28]. Greater parental insomnia symptoms were associated with greater sleep disturbances among their children, including insomnia, longer sleep onset latency, night awakenings, and wake time after sleep onset [24, 26, 29]. A major factor contributing to this worsening of parental sleep was COVID-19-related parental worries and stress [19, 29]. Although the pandemic’s impact on stress within families has been documented [30–32], research remains limited and mixed on the relationships between pandemic-specific family distress, parent sleep, and IS [17, 18, 20]. Prior literature reported that parental stress during the pandemic was not found to be related to parent-reported IS problems [19] and uninterrupted IS duration [22], yet linked to longer parent-reported IS latency [22]. Furthermore, one mediation analysis (*n* = 1355 parent–child dyads) indicated that COVID-19-related stress and worries in parents were indirectly linked to increased children’s sleep problems through parental insomnia symptoms [29].

The current state of knowledge regarding the associations between COVID-19-related family impact/stress and infant temperament is inconclusive. Several cross-sectional studies indicated that greater COVID-19-related life disruptions and postnatal stress were linked to infant temperament, including higher infant negative affectivity [5, 33], and lower surgency (a child’s capacity to be generally happy, active, and seeking stimulation) and self-regulation at 6 months [5, 34]. By contrast, in a longitudinal study (*n* = 175), findings indicated a nonsignificant relationship between the pandemic’s stress and temperament in infancy and early childhood [35], suggesting that the relationship may be dependent on other factors acutely and may diminish over time.

Given that new COVID-19 variants continue and other future infectious diseases may lead to restrictive public health measures, it is critical to investigate these relationships [36]. Based on the transactional model of IS, this study aimed to examine the relationships between the family impact and distress from the COVID-19 pandemic, parent insomnia, infant temperament, and parent-reported IS. We hypothesized that greater familial impact and distress related to COVID-19 pandemic stressors would be related to poorer parent-reported IS and mediated by greater parent-reported insomnia symptom severity and/or higher infant temperamental negative affectivity.

## Methods

### Study design and sample

In this cross-sectional study, 70 parents of infants from the Phoenix metropolitan area were recruited between February 27, 2021, and August 7, 2021. Recruitment sources included paid social media advertisements, local institutional email listservs, and website banner advertisements. Participants completed a 45-minute, REDCap-administered survey. During this period, the 7-day average of new reported cases in Arizona ranged from 1408 to 2228 [37]. The study was approved by the Institutional Review Board of Arizona State University, and electronic consent was provided.

The inclusion criteria were local mothers or fathers who had generally healthy infants up to 12 months old that were born full-term (≥37 weeks). Participants were excluded if (1) their infant had any congenital/genetic abnormalities (e.g. Down syndrome), developmental delays, neurological disorders (e.g. cerebral palsy), or other major birth complications; (2) they lived separately from the infant; or (3) the mother was currently experiencing significant postpartum complications.

## Measures

Parents reported parent and infant ages, parent and infant sex (female/male), parent race (American Indian/Native American, Asian, black, mixed race/other, Pacific Islander/Native Hawaiian, or white), education (<bachelor degree, bachelor degree, or above), marital status (single or married/in a domestic partnership), household income in US dollars (<$70 000, $70 000 and above, or do not want to share) [38], current feeding mode (exclusive breastfeeding or bottle feeding/mixed feeding), whether working from home currently (yes or no), and whether experiencing reduced working hours (yes or no).

The COVID-19 Exposure and Family Impact Survey (CEFIS) is a measure that assesses how the COVID-19 pandemic impacts families [39]. The CEFIS includes three primary scales: Exposure, Impact, and Distress [36]. The Exposure scale assesses the exposure to COVID-19 and related events (25 items with binary responses [yes/no], e.g. family exposure to someone with COVID-19, a family member hospitalized for COVID-19) [39]. The Impact scale (10 items, range: 1–4, cutoff: 2.5) is a four-point Likert scale ranging from 1 to 4 (a lot better; a little better; a little worse; a lot worse; and not applicable) to examine the impact of the COVID-19 pandemic stressors on family life (e.g. parenting, emotional well-being, physical well-being including eating, exercise, and sleeping), with higher scores indicating greater negative pandemic family impact [36, 39]. The Impact scale is recommended to not be scored if more than any 3 item responses are missing or marked “not applicable.” The Distress scale (2 items, range: 1–10, cutoff: 5) assesses the distress level that caregivers and their children have experienced due to the pandemic (e.g. overall distress you experienced related to COVID-19, stress your children experienced related to COVID-19), with a 10-point scale from 1 (no stress) to 10 (extreme distress) and with higher scores indicating greater pandemic family distress [39]. By dichotomizing at the cutoff, a score above 5 indicates negative valence (negative valence if >5, more distressed; positive valence if ≤5, less distressed) [36, 39]. Cronbach’s alphas for Impact and Distress scales were 0.92 and 0.76, respectively [36, 39]. The CEFIS has been validated among parents [39]. In this study, only the Impact and Distress scales were assessed.

The ISI (7 items, range: 0–28) measured self-perceived sleep disturbances and related distress of the target parent over the past 2 weeks. Each item is assessed using a five-point Likert scale, with responses ranging from “no problem” to “very severe.” Cronbach’s alpha was 0.74 among parents [40]. A cutoff score of 10 is meaningful in the general community [41].

The Infant Behavioral Questionnaire-Revised (IBQ-R, 37 items) was used to assess inherent reactivity and self-regulation in infancy [42]. Parents reported the frequency of each behavior during the last week on a seven-point Likert scale, from never to always. The IBQ-R consists of three subscales, including Surgency/Extraversion (13 items, a child’s capacity to be generally happy, active, and seeking stimulation), Negative Affectivity (12 items, high loadings on sadness, fear, and the degree to which a child is not easily calmed), and Effortful Control/Self-regulation (12 items, a child’s capacity to focus attention, avoid distractions, and employ planning) [5, 43]. Items within each scale are averaged to obtain the subscale scores. The subscales have demonstrated adequate internal consistency (Cronbach’s alpha = 0.71–0.90) [42]. In this sample, Cronbach’s alpha for three subscales were 0.87, 0.80, and 0.73, respectively. Given its stronger relationship with IS disturbance [11], only the Negative Affectivity subscale was analyzed in the present study.

The Brief Infant Sleep Questionnaire-Revised (BISQ-R, 33 items, range: 0–100) is a well-validated questionnaire that evaluates, over the past 2 weeks, the sleep patterns, sleep ecology, and parental perceptions of infants and toddlers, age 0–36 months [44, 45]. The BISQ-R consists of three subscales, including Infant Sleep (e.g. sleep onset latency, number and duration of night wakings, longest stretch of sleep, and total night sleep), Parent Perception (e.g. caregiver perceptions of bedtime difficulty, overnight sleep, and overall child sleep problems), and Parent Behavior (PB, e.g. bedtime, bedtime routine consistency, and parental behavior at the time of sleep onset and following night wakings) [44]. Higher scores indicate better IS quality, more positive IS perception, and parent behaviors promoting healthy and independent IS [44]. The average of the three subscale scores yields the total score.

## Data Analyses

SPSS statistical software Version 28 was used to obtain descriptive statistics, and Mplus software version 8.10 [46] was used to estimate bivariate correlations and conduct path analyses. The hypothesized path models were constructed based on the transactional model of IS [1]. The models specified that COVID-19 familial impact and/or distress (CEFIS Impact and Distress scales) directly and indirectly affect IS (BISQ-R total scale and IS subscale) through parental insomnia symptoms (ISI) and/or infant temperament (IBQ-R). We included both CEFIS Impact and Distress as antecedent variables. For Supplementary Analyses, we included only CEFIS Distress as the antecedent variable due to the strong correlation between CEFIS Impact and Distress (*r* = .51). Infant age, parent age, current feeding mode (exclusive breastfeeding vs. not), and whether experiencing reduced working hours (yes vs. no) were included as covariates. The bivariate correlations were obtained with maximum likelihood estimation and standard errors that are (1) robust to violations of normality and (2) yield optimal (i.e. unbiased) parameter estimates when data are incomplete [47]. Maximum likelihood estimation was also used to obtain standardized values for each path estimate and treat missing data (e.g. a latent variable missing data approach used for CEFIS Impact scale). The 95% parametric percentile bootstrap confidence intervals were used to test effects in the mediation models (path estimates and indirect effects), with 20 000 bootstrap samples used. Note that these confidence intervals do not assume data are normally distributed and are particularly recommended for mediation analysis, given that indirect effects are inherently non-normal [48–50], as each indirect effect is computed as a *product* of parameter estimates. We also estimated *R*^*2*^ values for each outcome and mediator using the Wald *z* test to assess these for significance. A sensitivity test, removing the sleep item from the CEFIS Impact scale, was also conducted for the mediation analyses. The findings remained the same.

## Results

Table 1 displays the sample’s descriptive characteristics. The average ages for parents and infants were 31.7 ± 5 years and 5.5 ± 3.5 months, respectively. The parent sample was predominantly female, identified as white, had obtained a bachelor degree or above, and was married or in a domestic partnership. Slightly more than half reported household incomes greater than $70K. The mean of CEFIS Distress was 4.57 (SD: 2.2). More than one-third (35.7%) of parents reported that their family experienced higher distress due to the pandemic (cutoff: 5). More than one-third reported meaningful insomnia symptom severity (ISI > 10). The average of IBQ-R negative affectivity, BISQ-R IS subscale score, and BISQ-R total score were 4.1 (SD: 1.1), 66.0 (SD:17.7), and 68.8 (SD: 12.7), respectively. The participants provided complete data for all the variables except household income, of which six participants (8.6%) did not provide data, and CEFIS Impact, of which 17 participants (24.3%) did not provide responses, because more than any three item responses were marked “not applicable.” Based on the demographics collected in the study, these missing individuals were not significantly different from the rest of the sample (chi-squared tests).

**Table 1.** Descriptive Characteristics of Parents and Infants (*N* = 70)

Sociodemographic characteristics *n* (%) or mean (SD)	
Parent age (mean, SD, years)	31.7 (5.0)
Infant age (mean, SD, months)	5.5 (3.5)
*Parent sex*	
Female	66 (94.3)
Male	4 (5.7)
*Infant sex*	
Female	29 (41.4)
Male	41 (58.6)
*Parent race*	
*Others*	*19 (27.1)*
American Indian/Native American	1 (1.4)
Asian	1 (1.4)
Black	0 (0)
Mixed race/others	17 (24.3)
Pacific Islander/Native Hawaiian	0 (0)
White	51 (72.9)
*Education*	
*Bachelor*	*20 (28.6)*
*Graduate*	*30 (42.9)*
Below bachelor degree	20 (28.6)
Bachelor degree or above	50 (71.4)
*Marital status*	
Single	1 (1.4)
Married or a domestic partnership	69 (98.6)
*Household income (USD)*	
<$70 000	24 (34.3)
$70 000 and above	40 (57.1)
Do not want to share	6 (8.6)
*Current feeding mode*	
Exclusive breastfeeding not breastfeeding	15 (21.4)
Bottle feeding or mixed feeding	55 (78.6)
*Working from home currently*	
*No*	*29 (41.4)*
*Not working before the pandemic*	*16 (22.9)*
Yes	25 (35.7)
No	45 (64.3)
*Whether experiencing reduced working hours*	
Yes	21 (30)
No	49 (70)
*COVID-19-related family stress, infant, and parental factors*	*n (%) or mean (SD)*
CEFIS Impact (range: 1–4, cutoff: 2.5)	2.72 (0.6)
CEFIS Distress (range: 1–10, cutoff: 5)	4.57 (2.2)
CEFIS Distress score > 5	25 (35.7)
ISI (range: 0–28)	8.7 (5.2)
ISI score > 10	25 (35.7)
IBQ-R negative affectivity (range: 1–7)	4.1 (1.1)
BISQ-R infant sleep subscale score (range: 0–100)	66.0 (17.7)
BISQ-R total score (range: 0–100)	68.8 (12.7)

CEFIS, COVID-19 Exposure and Family Impact Survey; ISI, Insomnia Severity Index; IBQ-R, Infant Behavioral Questionnaire-Revised; BISQ-R, Brief Infant Sleep Questionnaire-Revised.

### Bivariate associations between COVID-19 family impact and distress, parental insomnia symptoms, infant temperament, and parent-reported IS

Supplementary Table 1 shows the bivariate correlations among study variables in the path models. The poorer BISQ-R total score was related to greater infant negative affectivity (*r* = −.43, *p* < .001) and greater parental insomnia symptoms (*r* = −.32, *p* = .008). Lower parent-reported IS subscale scores were associated with greater infant negative affectivity (*r* = −.26, *p* = .033). Greater COVID-19 family distress was linked to greater infant negative affectivity (*r* = .40, *p* < .001) and greater parental insomnia symptoms (*r* = .28, *p* = .015). Greater COVID-19 family impact was associated with greater parental insomnia symptoms (*r* = .24, *p* = .033). COVID-19 family impact and family distress were not significantly related to the BISQ-R total score and IS subscale scores.

### Mediation effects of COVID-19 family impact and distress on IS through parental insomnia symptoms and infant temperamental negative affectivity

**Figure 1.**
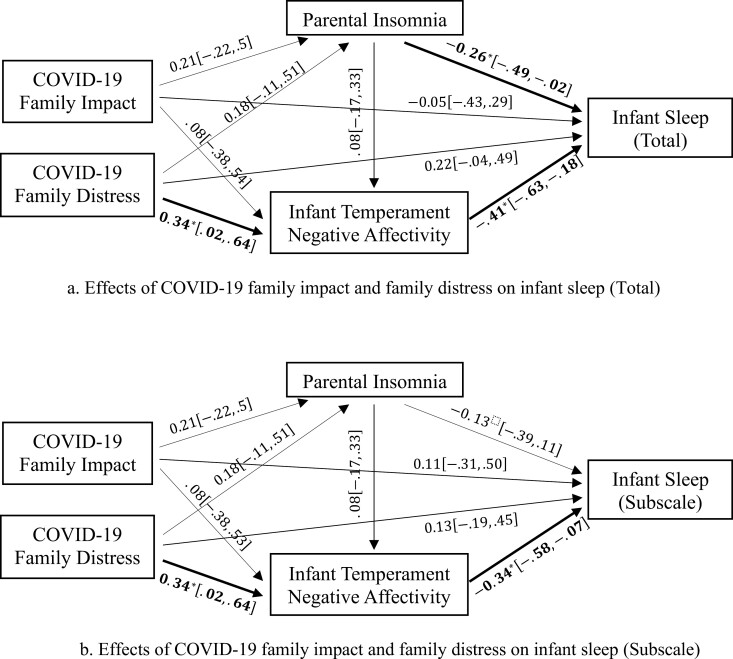
Path models for the effects of COVID-19 family impact and distress on infant sleep.

Figure 1 displays the standardized path estimates for the mediation models. We examined path models that include both COVID-19 family impact and distress in the same model. Figure 1 shows that both COVID-19 family impact and distress were not related to BISQ-R total and IS subscale scores directly in any model. However, family distress was positively related to infant negative affectivity, which was negatively associated with BISQ-R total and IS subscale scores (Figure 1, a and b). Furthermore, parental insomnia was related to BISQ-R total score, but not to IS subscale outcome. Additionally, we also examined path models that include only COVID-19 Distress as the antecedent variable (Supplementary Figure 1). The path estimates in Supplementary Figures 1, a and b also showed that infant negative affectivity mediated the relationships between COVID-19 family distress, and BISQ-R total and IS subscale scores.

In Table 2, both path models demonstrated that family distress during the pandemic was negatively associated with IS indirectly through infant temperament negative affectivity, including BISQ-R total score (β = −0.14, 95% CI [−0.32, −0.01]) and IS subscale score (β = −0.12, 95% CI [−0.27, −0.01]). Covariates, including parent age, infant age, whether experiencing reduced working hours, and current feeding mode, were not directly associated with any of the variables for either path analysis, except that current feeding mode was negatively related to IS subscale (β = −0.539, 95% CI [−1.114, −0.033]). Note that the variables in these mediation models accounted for 32% of the variation in BISQ-R total outcome (Wald *z* test = 3.70, *p < *.001), 20% of the variation in IS subscale (Wald *z* test = 2.45, *p = *.014), 23% of the variation in negative affectivity (Wald *z* test = 2.25, *p = *.024), and 13% of the variation in parental insomnia (Wald *z* test = 1.47, *p = *.142).

**Table 2.** Direct, Indirect, and Total Effects of COVID-19 Family Impact and Distress on Infant Sleep Via Parental Insomnia Symptom Severity and Infant Negative Affectivity

Variables	β (Bootstrap SE) [Bootstrap 95% CI]				
	Direct effect	Indirect effect via parental insomnia	Indirect effect via infant negative affectivity	Indirect effect via parental insomnia and infant negative affectivity	Total effect
COVID-19 family impact to BISQ-R total score	−0.05 [−0.43 to 0.29]	−0.05 [−0.18 to 0.05]	−0.03 [−0.23 to 0.16]	−0.01 [−0.05 to 0.02]	−0.14 [−0.55 to 0.26]
COVID-19 family distress to BISQ-R total score	0.22 [−0.04 to 0.49]	−0.05 [−0.18 to 0.02]	**−0.14** **[−0.32 to −0.01]**	−0.01 [−0.04 to 0.02]	0.03 [−0.29 to 0.36]
COVID-19 family impact to infant sleep subscale score	0.11 [−0.31 to 0.50]	−0.03 [−0.13 to 0.03]	−0.03 [−0.20 to 0.13]	−0.01 [−0.04 to 0.01]	0.05 [−0.39 to 0.47]
COVID-19 family distress to infant sleep subscale score	0.13 [−0.19 to 0.45]	−0.02 [−0.13 to 0.03]	**−0.12** **[−0.27 to −0.01]**	−0.01 [−0.05 to 0.02]	−0.01 [−0.34 to 0.33]

Effects adjusted for infant age, parent age, whether experiencing reduced working hours (yes vs. no), and current feeding mode (exclusive breastfeeding vs. not).

## Discussion

To the best of our knowledge, the present study is one of the few to investigate the relationships between the COVID-19 pandemic family impact and related stress and IS. In our sample, the findings indicated that more than one-third (35.7%) of the parents reported meaningful insomnia symptom severity (ISI > 10) and that their family experienced higher distress due to the pandemic (cutoff: 5). Part of our original hypothesis was confirmed that greater family distress was associated with poor IS indirectly through greater infant temperamental negative affectivity. However, parental insomnia symptom severity did not mediate the relationship between COVID-19 pandemic family distress and IS. Furthermore, family impact during the pandemic was not associated with IS directly and indirectly through parental insomnia symptom severity or infant temperamental negative affectivity. These results are partially consistent with the transactional model of IS [1], showing that IS may be partly predicted by both family context and infant factors.

During the pandemic, heightened stress levels in families with children were found in several studies [30–32]. Nearly one in four mothers of infants reported significantly high levels of stress (Perceived Stress Scale ≥25) [19]. Based on a national database of childcare closures in the United States [51], two-thirds of childcare centers closed in April 2020, while one-third remained closed in April 2021. As a consequence of childcare closures, parents were confronted with increased childcare demands while balancing work and life [52, 53]. Furthermore, social isolation and financial load were also significantly related to greater family stress [54].

The evidence on the direct association between COVID-19 pandemic family distress and parent-reported IS is mixed. Along with some research [19, 22], the current study found that COVID-19 pandemic family distress was not directly related to parent-reported IS problems, whereas other prior research found increases in pandemic-related parental stress were related to poor parent-reported IS quality and longer IS latency [21, 22]. Furthermore, studies conducted before the pandemic also found mixed associations between greater parental stress and parent-reported IS, including a decrease in the percentage and greater variability of nocturnal sleep [55], shorter daytime sleep duration and greater nocturnal wakefulness [2], and longer daytime sleep duration [9]. The relationship may be potentially affected by other factors, such as sample sizes, regions, age of infants, or timing of assessment during the pandemic. For example, the study aligned with our findings was conducted in the United States among infants aged 4 months (*n* = 149) in March 2020 [19]. In contrast to our findings, the other study was conducted among 452 babies aged from 0 to 35 months and 412 preschool children aged from 36 to 71 months from Europe during April and June 2020 [21].

Consistent with the transactional model of IS, we found that greater infant temperamental negative affectivity mediated the relation between greater COVID-19 pandemic family distress and poor parent-reported IS, including BISQ-R total score and the IS subscale (IS quality, e.g. sleep onset latency, number and duration of night wakings, longest stretch of sleep, and total night sleep). More specifically, distressed parents during the pandemic were more likely to perceive their infants as having higher levels of negative affectivity, which was associated with more parent-reported overall IS problems and worse IS quality. Similarly, a previous study also showed that infant temperament might serve as a possible mediator in the relationship between maternal stress and parent-reported IS problems [55].

The current studies on the relationship between stress specifically related to the COVID-19 pandemic and infant temperament are mixed and limited [5, 35]. Before the pandemic, there was already substantial evidence positively associating parental stress and infant temperamental negative affectivity in cross-sectional and longitudinal studies [56–60]. Our findings provided further evidence for a mediation pathway from COVID-19 family stress to IS through infant temperament. One possible explanation is that maternal stress during the pandemic can be transferred to infants through cortisol in breast milk, which, in turn, is positively linked to infant negative affectivity [61]. It seems more likely that maternal stress simply leads mothers to perceive more temperamental difficulty in their infants. Furthermore, early neurodevelopment in infancy is significantly influenced by the quality of parent–child interactions [62, 63]. COVID-19 pandemic family stress may negatively affect parent–infant interactions [62–64], and parents engage in fewer positive parenting practices [65], which may further shape infant temperamental negative affectivity [62–64].

In accord with our findings, the association between higher infant negative affectivity and poor parent-reported IS has already been cross-sectionally and longitudinally demonstrated, including poor sleep quality [6], shorter night sleep duration [66, 67], longer sleep latency [10, 11], and more acute and future night wakings [9, 10, 12]. This finding should be emphasized for a number of reasons. First, infants with higher negative affectivity tend to undergo negative emotions more intensely and frequently [68], which relate to poor sleep quality and shorter sleep durations [69]. Second, the predisposition to respond to negative emotions in infancy was found to be associated with greater difficulty in self-soothing [68]. This suggests that these infants have a lower capacity to fall asleep independently at the onset of a sleep period and when waking in the middle of the night [70]. They often require more intense comforting from their parents at bedtime and during the night; thus, their parents may be more inclined to rate their infant’s sleep as problematic [71]. Furthermore, the shared arousal regulatory processes in the central nervous system between infant temperament and sleep–wake behaviors might also account for the association [72].

Inconsistent with our hypothesis and the transactional model of IS, parental insomnia symptoms did not mediate the relationship between COVID-19 family distress and parent-reported IS (Figure 1). By contrast, other research found that among children aged from 9 to 17 years, COVID-19-related stress in parents was indirectly associated with increased parent-reported children’s sleep problems through parental insomnia symptoms [29]. The nonsignificant relation in our study may be related to the developmental stage of infancy. It is plausible that parental insomnia may function as a mediator of the relationship between COVID-19 family distress and child sleep problems at later points in development. In trying to further understand these relationships, we note that Supplementary Figure 1 indicated a positive relationship between COVID-19 family distress and parental insomnia. Previous studies have also found linkages between greater family stress and greater insomnia symptoms before and during the pandemic [29, 73–75]. It is noteworthy that family distress may lead one to hypervigilance, a state incompatible with sleep [76]. Specifically, COVID-19-related stressors could increase parental activity levels in the hypothalamic-pituitary-adrenal axis to produce the stress hormone cortisol [77, 78]. Under such circumstances, parents are more likely to become hypervigilant and experience difficulty returning to a state of relaxation, which is necessary for sleep onset and maintenance [79–85], contributing to greater insomnia symptom severity among parents.

There are limited studies examining the relationship between parental insomnia and IS during the pandemic. Of the few reported, which were mostly conducted in children but not infants, parental insomnia symptoms during the pandemic were positively related to parent-reported child sleep disturbances [24, 26, 29, 86]. Both Supplementary Figure 1 and Figure 1 found that parental insomnia symptoms were associated with BISQ-R total scale, but not IS subscale (IS quality, e.g. sleep onset latency, number and duration of night wakings, longest stretch of sleep, and total night sleep). More specifically, parents with greater insomnia symptoms were more likely to perceive their infant’s sleep as being problematic and experience worse parental bedtime behaviors with their infants, in line with other studies before the pandemic [87–89].

Inconsistent with our hypothesis, COVID-19 family impact was not associated with IS via infant temperamental negative affectivity. One possible reason could be the nonsignificant association between COVID-19 family impact and infant negative affectivity, as shown in Figure 1, suggesting that family life impact during the pandemic may not link to infant negative emotions and soothability directly (e.g. difficulty calming down and discomfort to limitations).

Several limitations in the current study should be acknowledged. First, this was a cross-sectional study. Both mediation analyses provided insight into the possible mechanistic relationship between COVID-19 pandemic disruptions and IS. However, future longitudinal data are needed to determine the directionality of the relationship. Second, all measures were rated by parents, increasing the potential for response bias. Future studies should consider involving objective and/or prospective measures, for example, actigraphy IS and sleep diaries, and cortisol level for parental distress to further test both models more thoroughly. Third, the sample size was relatively small and predominately white. Future research should include more diverse populations in a larger sample to generalize the findings to a broader population. Fourth, we included only one parental variable (insomnia) in this study. Fifth, this study examined only the total score on the BISQ-R, and the three subscales, but did not examine individual components (e.g. bedtime, night awakenings). We will involve more actigraphy data and parental variables, such as anxiety, depression, and distress, to further examine relationships in our future studies. Finally, the CEFIS Exposure scale was not used.

## Conclusion

This study was one of the few to investigate the relationships between family impact, family distress, and IS during the unique period of the COVID-19 pandemic. From our findings, we concluded that greater family distress due to COVID-19 pandemic stressors was associated with poorer IS through greater higher infant negative affectivity. Parental insomnia symptom severity was not a significant mediator. Interventions are needed to support family psychological health and healthy behaviors including sleep. Additional childcare support for parents with infants to relieve parental distress should be anticipated and planned carefully in case of future health crises. Effective and scalable public health education programs promoting nurturing environments, positive parenting-child interactions, and infant temperament development are also needed.

## Supplementary Material

zpae061_suppl_Supplementary_Material

## Data Availability

Materials described in the manuscript, including all relevant raw data, will be freely available to any researcher wishing to use them for noncommercial purposes, without breaching participant confidentiality.
