# Supplementary material for: The relationships between the family impact and distress of the coronavirus disease-19 pandemic, parent insomnia, infant temperamental negative affectivity, and parent-reported infant sleep: a path analysis
Source: Sleep Adv. 2024 Aug 14;5(1):zpae061. doi: 10.1093/sleepadvances/zpae061 (PMC11380114; doi:10.1093/sleepadvances/zpae061)
Supplement: zpae061_suppl_Supplementary_Material [file zpae061_suppl_supplementary_material.docx]

**The relationships between the family impact and distress of the COVID-19 pandemic, parent insomnia, infant temperamental negative affectivity, and parent-reported infant sleep: a path analysis**

Nana Jiao^a,^ MSN, RN, PhD; Keenan A. Pituch^a^, PhD; Megan E. Petrov^a^, PhD

^a^Edson College of Nursing and Health Innovation, Arizona State University, Phoenix, AZ, USA

**Address correspondence to:**

Nana Jiao, MSN, RN, PhD

Edson College of Nursing and Health Innovation

Arizona State University

500 N. 3^rd^ Street, Ste 301

Phoenix, AZ 85004

Phone: 602-475-9167

Email: [nana.jiao@asu.edu](mailto:nana.jiao@asu.edu)

**A list of captions for supplementary tables and figures**

Supplementary Table 1 Correlations for the Study Variables

Supplementary Figure 1 Path models for the effects of COVID-19 family impact on infant sleep

**Supplementary Table 1**

Correlations for the Study Variables

|  | 1 | 2 | 3 | 4 | 5 | 6 | 7 |
| --- | --- | --- | --- | --- | --- | --- | --- |
| 1 Overall Infant Sleep  (BISQ-R total score) |  |  |  |  |  |  |  |
| 2 Infant Sleep Subscale | **0.79^**^** |  |  |  |  |  |  |
| 3 Infant Negative Affectivity | **-0.43^**^** | **-0.26^*^** |  |  |  |  |  |
| 4 Parental Insomnia | **-0.32^**^** | -0.15 | 0.20 |  |  |  |  |
| 5 Infant Age | -0.18 | 0.14 | 0.22 | 0.03 |  |  |  |
| 6 Parent Age | -0.11 | 0.06 | 0.02 | -0.07 | 0.24 |  |  |
| 7 COVID-19 Family Impact | -0.14 | 0.06 | 0.31 | **0.24^*^** | 0.08 | 0.20 |  |
| 8 COVID-19 Family Distress | -0.10 | -0.04 | **0.40^**^** | **0.28^*^** | 0.03 | 0.10 | **0.51^**^** |

Note: ^*^p<0.05, ^**^p<0.01.

**Supplementary Figure 1**

Parental Insomnia

$$\mathbf{.29}^{\mathbf{*}}\left[ \mathbf{.02,.54} \right]$$

$$\boldsymbol{-.27}^{\boldsymbol{*}}\left[ \boldsymbol{-.48,-.04} \right]$$

Infant Sleep

(Total)

COVID-19

Family Distress

$$.09\left[ -.17,.34 \right]$$

$$.20 \left[ -.01,.42 \right]$$

$$\boldsymbol{-}\boldsymbol{.42}^{\boldsymbol{*}}\left[ \boldsymbol{-.62,-.21} \right]$$

$$\boldsymbol{.38}^{\boldsymbol{*}}\left[ \boldsymbol{.12,.61} \right]$$

Infant Temperament Negative Affectivity

a. Effects of COVID-19 family distress on infant sleep (Total)

$$-.11\left[ -.35,.12 \right]$$

Parental Insomnia

$$\mathbf{.29}^{\mathbf{*}}\left[ \mathbf{.02,.54} \right]$$

$$.18 \left[ -.04,.43 \right]$$

Infant Sleep

(Subscale)

COVID-19

Family Distress

$$.09\left[ -.17,.34 \right]$$

$$\boldsymbol{-}\boldsymbol{.33}^{\boldsymbol{*}}\left[ \boldsymbol{-.56,-.08} \right]$$

$$\boldsymbol{.38}^{\boldsymbol{*}}\left[ \boldsymbol{.12,.61} \right]$$

Infant Temperament Negative Affectivity

b. Effects of COVID-19 family distress on infant sleep (Subscale)

Supplementary Figure 1. Path models for the effects of COVID-19 family impact on infant sleep

Note: adjusted for infant age, parent age, whether experiencing reduced working hours, and current feeding mode
